# Supplementary material for: Contribution of Ezrin on the Cell Surface Plasma Membrane Localization of Programmed Cell Death Ligand-1 in Human Choriocarcinoma JEG-3 Cells
Source: Pharmaceuticals (Basel). 2021 Sep 24;14(10):963. doi: 10.3390/ph14100963 (PMC8540387; doi:10.3390/ph14100963)
Supplement: Supplementary file 1 [file pharmaceuticals-14-00963-s001.zip › pharmaceuticals-1341650-supplementary.pdf]

pharmaceuticals

Supplementary Materials

Contribution of Ezrin on the Cell Surface Plasma Membrane Localization of Programmed Cell Death Ligand-1 in Human Choriocarcinoma JEG-3 Cells

Mayuka Tameishi[1], Takuro Kobori[1,*], Chihiro Tanaka[1], Yoko Urashima[1], Takuya Ito[2], and Tokio Obata[1,*]

[1] Laboratory of Clinical Pharmaceutics, Faculty of Pharmacy, Osaka Ohtani University, Tondabayashi, 584-8540 Osaka, Japan; u4117083@osaka-ohtani.ac.jp (M.T.); u4117078@osaka-ohtani.ac.jp (C.T.); urasiyo@osaka-ohtani.ac.jp (Y.U.)
[2] Laboratory of Natural Medicines, Faculty of Pharmacy, Osaka Ohtani University, Tondabayashi, 584-8540 Osaka, Japan; itoutaku@osaka-ohtani.ac.jp
* Correspondence: koboritaku@osaka-ohtani.ac.jp (T.K.); obatatoki@osaka-ohtani.ac.jp (T.O.); Tel.: +81-721-24-9374 (T.K.); +81-721-24-9371 (T.O.)

Expression analysis of moesin mRNA in primary human umbilical vein endothelial cells (HUVEC) and HeLa cells.

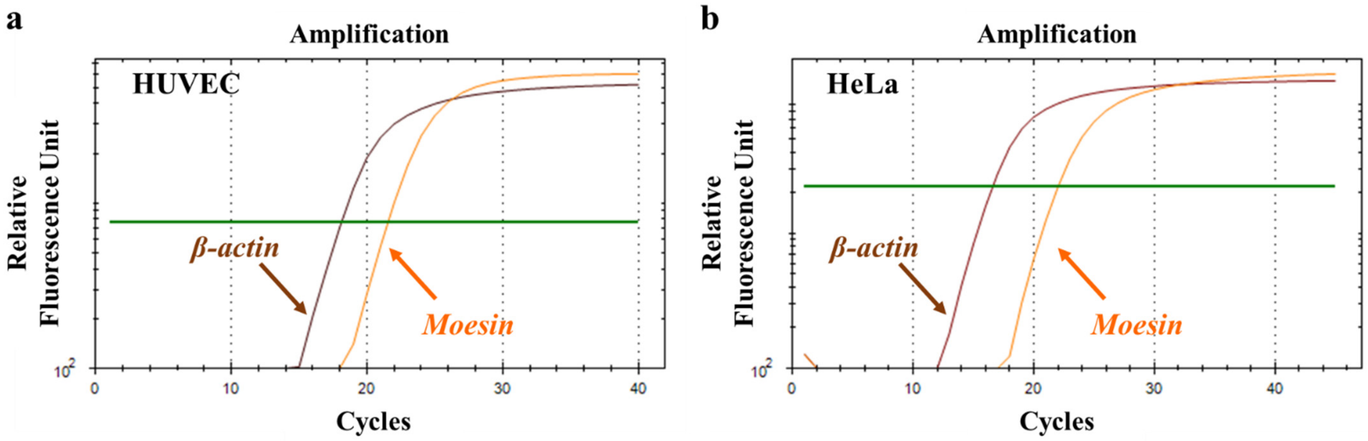

Figure S1. Expression analysis of moesin mRNA in primary human umbilical vein endothelial cells (HUVEC) and HeLa cells. Representative amplification curves of *moesin* together with *β-actin* (internal control) in **(a)** HUVEC and **(b)** HeLa cells, a human uterine cervix cell line, as determined by real-time quantitative reverse transcription-polymerase chain reaction.

Materials and Methods for Figure S1

Cell Culture

The primary human umbilical vein endothelial cell (HUVEC) was purchased from PromoCell (C-12208; Heidelberg, Germany) and were cultured in CS-C complete medium, a growth medium optimized for HUVEC (Cell Systems, Kirkland, WA, USA) supplemented with heat-inactivated 10% fetal bovine serum (FBS) (BioWest, Nuaillé, France). The human uterine cervix cell line, HeLa cells, were purchased from European Collection of Cell Cultures (ECACC) (EC93021013-F0; KAC, Hyogo, Japan) and were cultured in Dulbecco's modified Eagle medium (DMEM) containing 1,500 mg/L glucose (FUJIFILM Wako Pure Chemical, Osaka, Japan) supplemented with heat-inactivated 10% FBS (BioWest). Both cells were maintained until 70-80% confluent at 37°C in a humidified atmosphere with 5% CO_2.

Gene expression profile of programmed cell death ligand-1 (PD-L1), ezrin, radixin, and moesin in human choriocarcinoma cell lines.

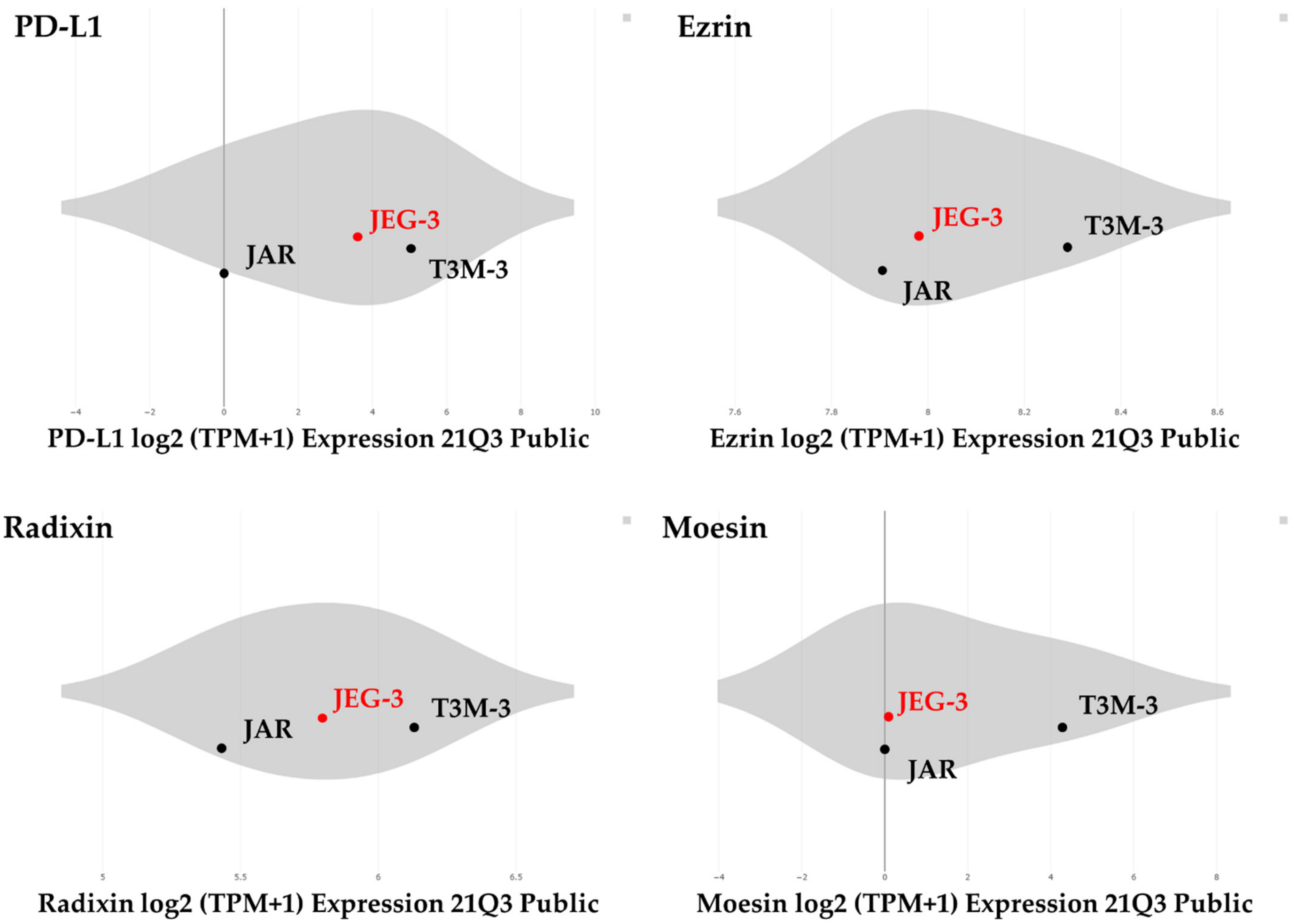

Figure S2. Gene expression profile of programmed cell death ligand-1 (PD-L1), ezrin, radixin, and moesin in human choriocarcinoma cell lines. Relative gene expression patterns of *PD-L1* in addition to *ezrin*, *radixin*, and *moesin* in three human choriocarcinoma cell lines, JAR, JEG-3, and T3M-3, registered in the database of Cancer Cell Line Encyclopedia (CCLE) were determined by utilizing the Cancer Dependency Map (DepMap) portal data explorer. Scatter plots showing the expression levels (log2 (TPM+1)) of each gene in human choriocarcinoma cell lines. Data from CCLE and DepMap were obtained from the 2021Q3 release.

Original western blotting images of programmed cell death ligand-1 (PD-L1), ezrin, radixin, and moesin as well as glyceralde-hyde-3-phosphate dehydrogenase (GAPDH) in JEG-3 cells.

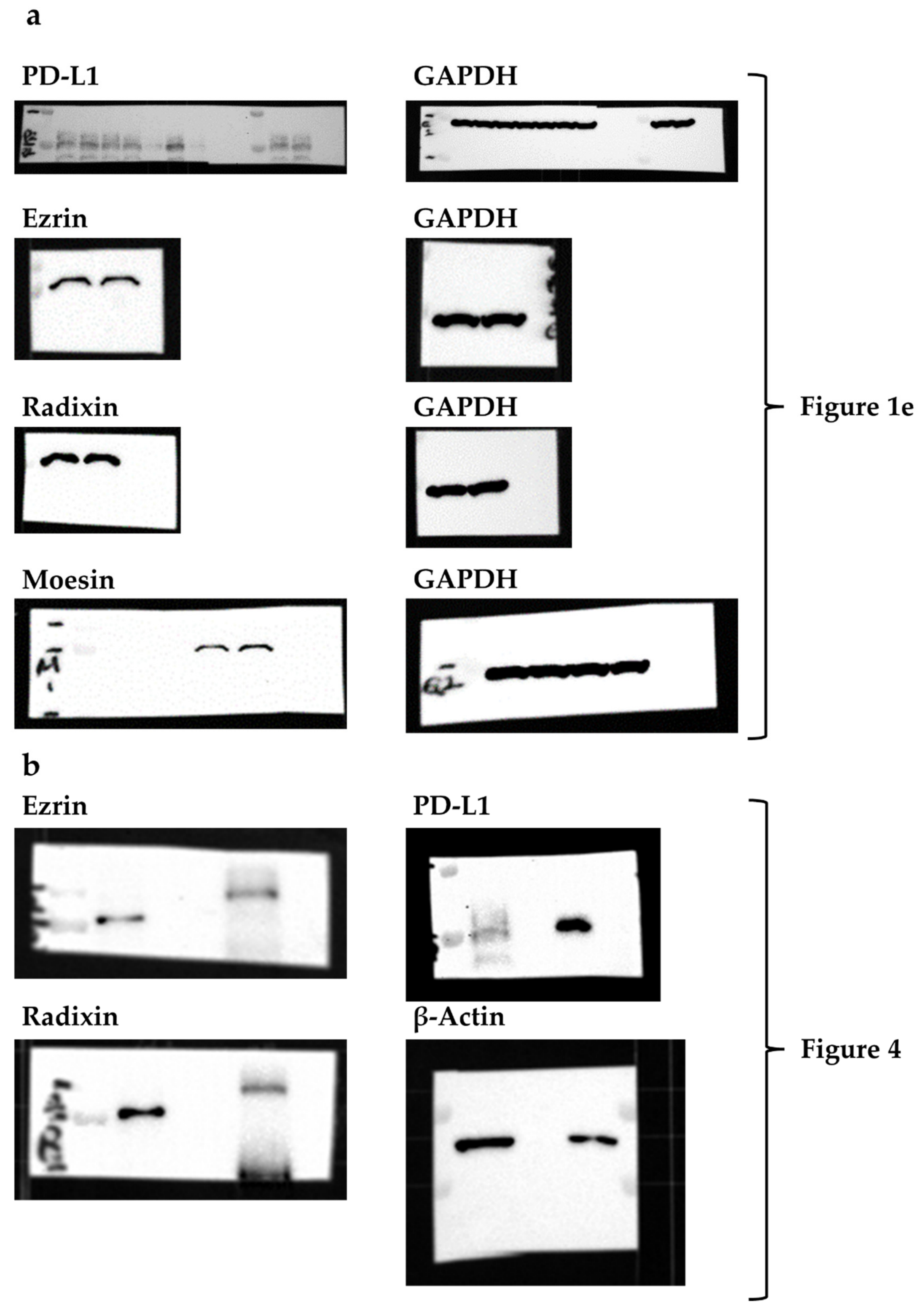

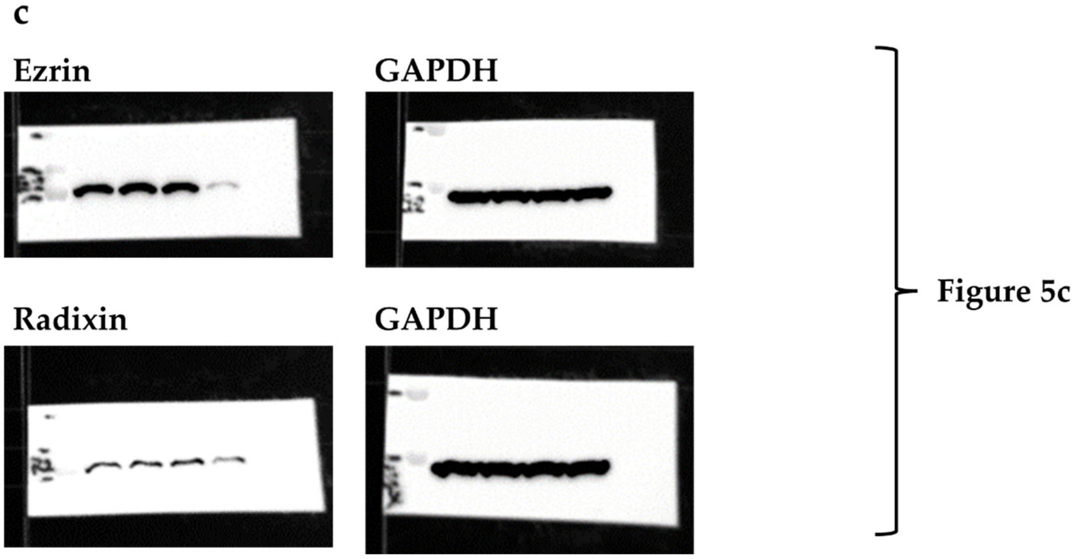

Figure S3. Original western blotting images of programmed cell death ligand-1 (PD-L1), ezrin, radixin, and moesin as well as glyceraldehyde-3-phosphate dehydrogenase (GAPDH) in JEG-3 cells. **(a)** The original Western Blotting membrane to confirm the protein expression of PD-L1, ezrin, radixin, and moesin shown in Figure 1e. **(b)** The original Western Blotting membrane to detect the protein-protein interaction between PD-L1 and ezrin, radixin as well as actin in the whole cell lysates (input) and those in the immunoprecipitates (IP) using a control antibody or an anti-PD-L1 antibody shown in Figure 4. **(c)** The original Western Blotting membrane to measure the protein expression levels of ezrin and radixin as well as GAPDH shown in Figure 5c.
